# Candidate MicroRNA Regulatory Axes in Melanoma CD8+ T Cell Exhaustion: A Network-Based In Silico Stratification

**DOI:** 10.7759/cureus.114850

**Published:** 2026-08-20

**Authors:** Muhammad Zain U Javed, Muhammad Hussain

**Affiliations:** 1 Immunology, Shifa International Hospitals Limited, Islamabad, PAK; 2 Immunology, Armed Forces Institute of Pathology, Rawalpindi, PAK

**Keywords:** anti-pd-1, cerna, immune checkpoint blockade, lncrna, melanoma, microrna, t cell exhaustion, tumor microenvironment

## Abstract

Background

Anti-programmed cell death protein-1 (anti-PD-1) immunotherapy has transformed the treatment of advanced melanoma, but durable benefit remains limited to a subset of patients. CD8+ T cell exhaustion contributes to immune escape in the melanoma tumor microenvironment, while the post-transcriptional regulation of exhaustion-associated genes by microRNAs (miRNAs) remains incompletely understood.

Objective

We characterized miRNA-mRNA regulatory associations in CD8+ T cell exhaustion-enriched melanoma transcriptomes, classified inverse associations as loss-of-repression (LoR) or active suppression (AS), extended the network to candidate long non-coding RNA (lncRNA)-miRNA-mRNA relationships, and examined whether network-derived transcriptomic scores were associated with anti-PD-1 outcomes in independent cohorts.

Methods

The TCGA-SKCM bulk transcriptomes were filtered by single-sample gene set enrichment analysis (ssGSEA), yielding 121 CD8+ T cell exhaustion-enriched cases and 115 cases with paired miRNA and mRNA measurements. Differentially expressed miRNAs (DEmiRNAs) were identified between fixed high- and low-exhaustion tertiles (n = 38 each) using two-sided Mann-Whitney U tests with Benjamini-Hochberg correction (|log₂FC| ≥ 0.5; false discovery rate (FDR) ≤ 0.05). Database-supported inverse Spearman's correlations (ρ ≤ -0.30; FDR ≤ 0.05) were assembled into a bipartite network. Edges were classified as LoR or AS, and a competing endogenous RNA (ceRNA) extension incorporated DIANA-LncBase/ENCORI lncRNA-miRNA interactions. Nine biologically anchored axes underwent continuous-score analysis and HC3 regression adjusted for tumor purity, CD8, fibroblast/CAF, myeloid, interferon-gamma, and sample type, together with four sensitivity analyses. Exploratory clinical testing used GSE78220 and the pre-PD-1 biopsy subset of the DFCI melanoma cohort (cBioPortal study identifier: mel_dfci_2019).

Results

Twenty-six DEmiRNAs (20 upregulated and 6 downregulated) formed 326 inverse miRNA-mRNA edges, comprising 263 AS and 63 LoR associations. Six of nine focused axes met the adjusted-support criterion, and all six were AS-classified including upregulated miR-155-5p as the dominant hub, with inverse associations involving FOXO3 (*ρ *= -0.305, adjusted p = 0.020) and MEIS1, the strongest priority edge (*ρ *= -0.470, adjusted p < 0.0001). NEAT1, MALAT1, and XIST emerged as the top-degree lncRNA hubs, all classified as AS-sponge type. Exclusion of one solid-tissue-normal specimen left 114 paired tumors, retained 25 DEmiRNAs, and supported seven of nine axes in the tumor-only sensitivity analysis. Relational integration with lncRNA-miRNA records yielded 29,274 candidate chains, including 790 containing a focused miRNA-mRNA edge. Transcriptomic score analyses were negative and exploratory in GSE78220 (mRNA proxy area under the curve (AUC) = 0.631; Mann-Whitney p = 0.269) and in the DFCI melanoma cohort (mRNA topology AUC = 0.453, p = 0.451; lncRNA topology AUC = 0.388, p = 0.0695).

Conclusions

The LoR/AS framework offers a transparent means of organizing correlative miRNA-mRNA hypotheses in melanoma bulk transcriptomes. The adjusted results favored an AS pattern among the focused axes, but they did not establish CD8+ T-cell-intrinsic regulation, direct miRNA targeting, ceRNA activity, or clinical predictive utility. Experimental testing in sorted or single-cell melanoma CD8+ tumor-infiltrating lymphocyte systems is required.

## Introduction

Melanoma is among the most immunogenic solid tumors, and immune checkpoint blockade has fundamentally changed its prognosis. Phase III trials of pembrolizumab and nivolumab demonstrated objective responses in approximately one-third to two-fifths of previously untreated patients [1,2]. Longer follow-up has confirmed that programmed cell death protein-1 (PD-1)-based treatment can produce durable survival, including with nivolumab alone or in combination with ipilimumab [3]. Nevertheless, primary resistance and relapse remain common, and no pre-treatment biomarker has achieved universal prospective clinical validation [4].

CD8+ T cell exhaustion is an important biological contributor to resistance within chronically stimulated tumors [4]. The exhausted state encompasses sustained expression of inhibitory receptors such as PD-1, T-cell immunoglobulin and mucin domain-containing protein 3 (TIM-3), lymphocyte-activation gene 3 (LAG-3), T-cell immunoreceptor with immunoglobulin and immunoreceptor tyrosine-based inhibitory motif domains (TIGIT), and cytotoxic T-lymphocyte-associated protein 4 (CTLA-4); diminished cytokine polyfunctionality and proliferative capacity; and progressive epigenetic stabilization [5]. Transcription factors, including thymocyte selection-associated high-mobility-group box (TOX), nuclear receptor subfamily 4 group A (NR4A) family members, early growth response 2 (EGR2), and basic leucine zipper transcription factor, ATF-like (BATF), help establish and maintain this program [6,7]. Exhaustion is also heterogeneous, e.g., progenitor-like TCF7+ or SLAMF6+ cells retain proliferative potential and may respond to PD-1 blockade, whereas more terminally differentiated states show restricted reinvigoration [8].

MicroRNAs are endogenous non-coding RNAs, typically 19-24 nucleotides long, that regulate transcript stability and translation through sequence-dependent interactions with target RNAs. Long non-coding RNAs (lncRNAs) and circular RNAs can add further layers of post-transcriptional regulation, including putative competing endogenous RNA (ceRNA) relationships [9]. Immune miRNAs have established roles in lymphocyte activation and inflammatory signaling; examples include miR-155 in inflammatory T cell development and miR-146 in nuclear factor (NF)-kappa B-dependent feedback regulation [10,11]. Previous melanoma studies have reconstructed ceRNA networks from public transcriptomes and related selected lncRNA hubs to disease progression or prognosis [12,13]. A network centered specifically on CD8+ T cell exhaustion may therefore provide a useful experimental prioritization framework, provided that bulk-tissue associations are not mistaken for cell-intrinsic mechanisms.

The primary objective of this study was to identify miRNAs associated with variation in a CD8+ T cell exhaustion program in TCGA-SKCM and to construct a database-supported miRNA-mRNA network. Secondary objectives were to distinguish LoR and AS directional patterns, evaluate nine biologically anchored axes after adjustment for major tumor-microenvironment covariates, extend the network to candidate lncRNA-miRNA-mRNA chains, and test downstream transcriptomic scores in two anti-PD-1 melanoma cohorts.

## Materials and methods

This retrospective in silico bioinformatics study used publicly available, de-identified transcriptomic and clinical data. No human participants were directly enrolled, and institutional ethical approval was not required. Paired miRNA expression, measured as reads per million (RPM), and mRNA expression quantified using RNA-Seq by Expectation-Maximization (RSEM; log₂-normalized) from the Cancer Genome Atlas Skin Cutaneous Melanoma cohort (TCGA-SKCM) were obtained via the cBioPortal REST API (n = 472 samples) (https://www.cbioportal.org) [14]. The immune-enrichment procedure selected 121 cases, of which 115 had paired miRNA and mRNA matrices. A specimen-level Genomic Data Commons (GDC) audit classified the 121 selected cases as 100 metastatic tumors, 20 primary tumors, and 1 solid-tissue-normal specimen (TCGA-GN-A4U8-11A). All 115 paired miRNA-mRNA samples were included in the primary analysis. A separate tumor-only sensitivity analysis excluded TCGA-GN-A4U8-11A, leaving 114 paired tumor samples. The pre-treatment anti-PD-1 melanoma cohort, GSE78220, was attributed to Hugo et al. and was accessed via the NCBI Gene Expression Omnibus (https://www.ncbi.nlm.nih.gov/geo/query/acc.cgi?acc=GSE78220) [15]; Pt16.OnTx was excluded, and the pre-treatment biopsies Pt27A and Pt27B were averaged, yielding 26 patients (14 responders and 12 non-responders). The miRNA-mRNA support was obtained from miRTarBase (https://mirtarbase.cuhk.edu/) [16] and miRDB v6.0 (https://mirdb.org) at a prediction score ≥ 80 [17]. The Liu et al. cohort was used for independent downstream transcriptomic testing [18]. All inferential tests were two-sided.

Single-sample gene set enrichment analysis (ssGSEA) was performed with GSEApy v1.1.13 (Zhuoqing Fang and contributors; Stanford, California, USA) using rank-based sample normalization [19]. Cases were retained when CD8+ T cell and CD8+ exhausted scores were at or above their cohort medians, and the fibroblast/cancer-associated fibroblast (CAF) score was at or below the 60th percentile. For covariate adjustment, specimen-matched tumor purity was represented by the consensus purity estimate (CPE) of Aran et al. [20], available for 120 of 121 selected cases and 114 of 115 paired cases. Myeloid infiltration was scored from the union of MCP-counter monocytic-lineage, myeloid-dendritic-cell, and neutrophil signatures [21]. Interferon-gamma inflammation was represented by the MSigDB Hallmark Interferon Gamma Response gene set [22].

High- and low-exhaustion groups were defined as the upper and lower tertiles of the CD8⁺ T-cell exhaustion ssGSEA score within the retained TCGA-SKCM subset. DEmiRNA log2 fold change was calculated as the difference in the mean normalized log2 expression using a two-sided Mann-Whitney U test, corrected by the Benjamini-Hochberg (BH) method; DEmiRNAs required |log_2_FC|≥0.5, false discovery rate (FDR) ≤ 0.05, and expression in at least 80% of high-exhaustion samples. Candidate miRNA-mRNA pairs were retained when Spearman's rho was ≤-0.30, edge FDR was ≤0.05, and the interaction was represented in miRDB and/or miRTarBase. LoR denoted a downregulated miRNA with an inverse target association, whereas AS denoted an upregulated miRNA with an inverse target association. These labels describe directionality and do not establish a molecular mechanism. Bipartite density was calculated as the number of edges divided by the product of miRNA and mRNA node counts.

Continuous and covariate-adjusted analyses were used to examine whether the grouped findings were robust to exhaustion-score variation and tissue composition. For every network edge, miRNA and target expression were correlated separately with the continuous exhaustion score. Nine axes selected for focused biological evaluation were then modeled with standardized target-mRNA expression as the outcome and standardized miRNA expression as the predictor, adjusting for CPE purity, CD8 total, fibroblast/CAF, myeloid, interferon-gamma, and primary-versus-metastatic sample type. Regression with MacKinnon-White HC3 heteroskedasticity-consistent standard errors (HC3) and residual rank-based partial correlations were calculated in 114 complete cases. An axis met the exploratory adjusted-support criterion when beta remained negative, nominal p ≤ 0.05, and BH FDR ≤ 0.10 across the nine models. Sensitivity analyses used quartile extremes, leave-one-out deletion, exclusion of the non-tumor specimen, and an independent gate based on MCP-counter CD8/cytotoxic and fibroblast signatures with a compact exhaustion core. The independent gate retained 96 cases, including 90 paired cases, and overlapped the primary gate in 86 cases (Jaccard index = 0.6565).

lncRNA-miRNA interactions for all 26 exhaustion-associated DEmiRNAs were retrieved from DIANA-LncBase v3 (https://diana.e-ce.uth.gr/lncbasev3/interactions) and Encyclopedia of RNA Interactomes (ENCORI/starBase)(https://rnasysu.com/encori). Interactions supported by both databases were prioritized, and all non-duplicate pairs were combined. These interactions were integrated with the miRNA-mRNA network to construct lncRNA→miRNA⊣mRNA regulatory pathways (where "→" denotes the database recorded lncRNA-miRNA interaction and "⊣" denotes the inverse, repressive miRNA-mRNA correlation), representing potential ceRNA-mediated sponge effects. Priority ceRNA pathways were defined as those containing a priority miRNA-mRNA edge, and lncRNA network topology metrics (degree, weighted degree, betweenness centrality, eigenvector centrality) were evaluated using NetworkX (v3.6.1) (https://networkx.org). Hub lncRNA expression was tested for exhaustion-stratified differential expression in the lncRNA hub set using the same 121 retained CD8/exhaustion-enriched TCGA-SKCM samples and the same high-versus low tertile definitions.

For GSE78220, a priority-target mRNA proxy score was computed as mean z-scored protective targets (FOXO3, MEIS1, ITPKB, MAVS, PIK3CA) minus mean z-scored exhaustion-associated targets (PDCD1, EGR2, NR2F6, ZEB2) since direct miRNA scoring was not possible because the GSE78220 miRNA matrix was unavailable locally. For the Liu et al. 2019 cohort, log2(TPM+1) RNA-seq expression values were z-scored across patients, and four transcriptomic scores were evaluated: connected mRNA topology hubs, the function-minus-exhaustion mRNA axis, top available lncRNA hubs, and the four-lncRNA priority hub score. Progression-free survival (PFS) values came from a secondary published-supplement-derived table matched to Hugo patient identifiers rather than directly from GEO, so PFS testing was designated ancillary. The receiver operating characteristic (ROC) area under the curve (AUC) was accompanied by 2,000 nonparametric bootstrap resamples; score distributions were compared by two-sided Mann-Whitney U tests, and median splits were used for exploratory Kaplan-Meier and log-rank analyses. Analyses were run in Python (v3.13.11) (Python Software Foundation; Wilmington, Delaware, USA) with pandas (v2.3.3; pandas Development Team/NumFOCUS, Austin, Texas, USA), NumPy (v2.4.4; NumPy Developers/NumFOCUS, Austin, Texas, USA), SciPy (v1.17.1; SciPy Developers/NumFOCUS, Austin, Texas, USA), statsmodels (v0.14.6; statsmodels Development Team), NetworkX (v3.6.1; NetworkX Developers/NumFOCUS, Austin, Texas, USA), scikit-learn (v1.8.0; scikit-learn community/Inria-hosted consortium, Le Chesnay-Rocquencourt, France), and lifelines (v0.30.3; Cameron Davidson-Pilon and contributors, Toronto, Ontario, Canada). Figures were generated with Matplotlib (v3.10.8; Matplotlib Development Team/NumFOCUS, Austin, Texas, USA) and seaborn (v0.13.2; Michael Waskom and contributors, New York, New York, USA).

## Results

**Figure 1:**
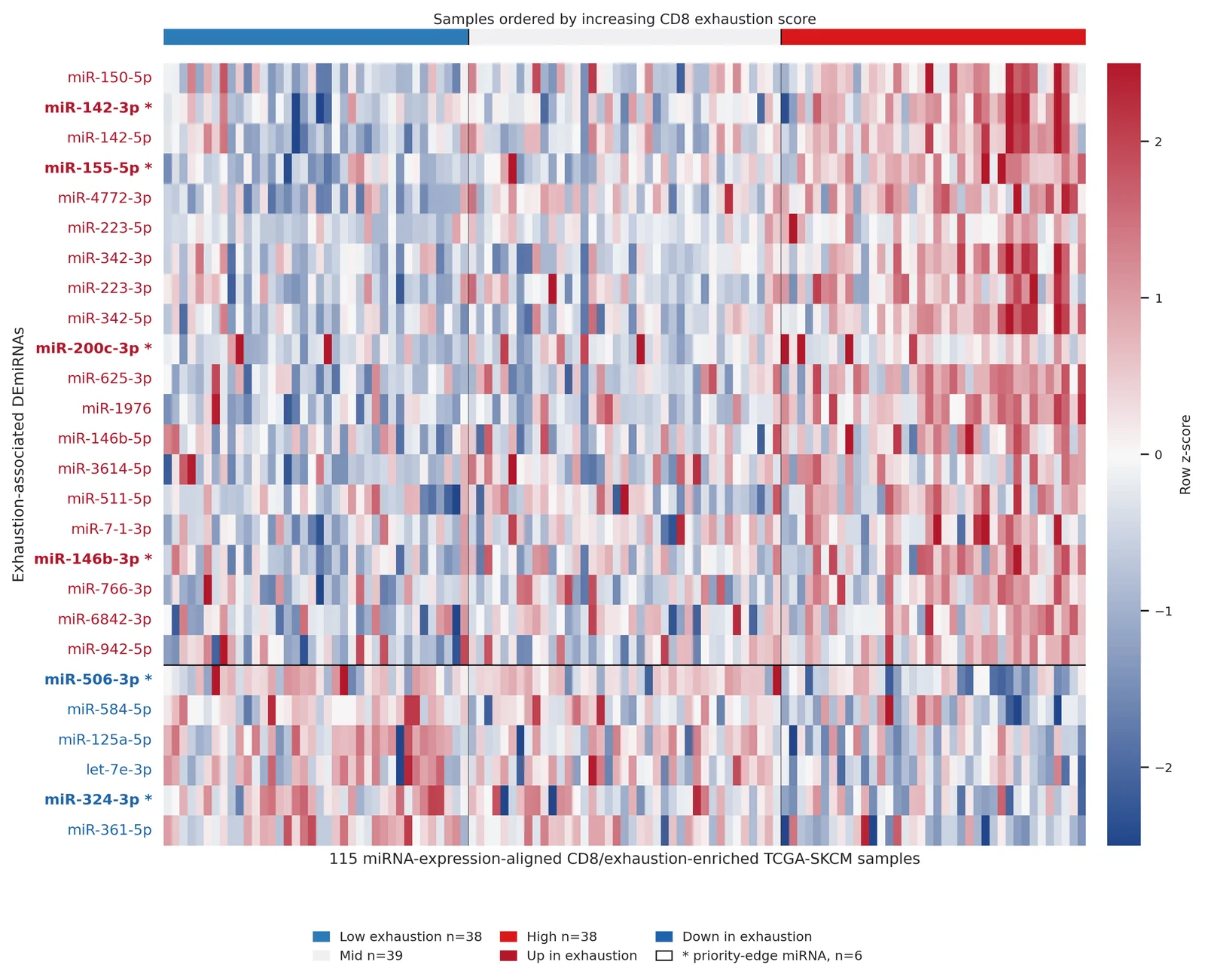
Heatmap of exhaustion-associated DEmiRNA expression in CD8/exhaustion-enriched TCGA-SKCM samples. 115/121 CD8-enriched samples had matched miRNA expression. TCGA-SKCM: Cancer Genome Atlas Skin Cutaneous Melanoma; DEmiRNA: differentially expressed microRNA

The ssGSEA filtering retained 121 TCGA-SKCM samples enriched for CD8/exhaustion signatures and relatively depleted for fibroblast signatures. Differential miRNA expression analysis between high- (n = 38) and low-exhaustion (n = 38) tertile groups identified 26 DEmiRNAs using normalized log2 expression mean differences (20 upregulated and 6 downregulated in high-exhaustion samples) (Figure 1).

Among upregulated miRNAs, the three most significantly dysregulated were hsa-miR-155-5p (log_2_FC=+1.44, FDR=1.79x10^-6^), hsa-miR-1976 (log_2_FC = +0.93, FDR = 1.79 x 10^-6^), and hsa-miR-142-5p (log_2_FC = +1.46, FDR = 1.57 x 10^-5^). hsa-miR-150-5p displayed the largest fold change among upregulated miRNAs (log_2_FC = +1.89). Among downregulated miRNAs, hsa-miR-506-3p showed the most dramatic reduction (log_2_FC = -2.34, FDR = 0.017). hsa-miR-584-5p (log_2_FC = -0.90) and hsa-miR-125a-5p (log_2_FC = -0.77) were also significantly downregulated. Several upregulated miRNAs belonged to established hematopoietic or immune-regulatory families, including miR-155, miR-150, miR-142, miR-146, miR-342, and miR-223 (Table 1).

**Table 1:** Selected exhaustion-associated DEmiRNAs in CD8/exhaustion-enriched TCGA-SKCM samples. FDR values are BH-adjusted p-values. The 26-miRNA list is provided in the Appendices. TCGA-SKCM: Cancer Genome Atlas Skin Cutaneous Melanoma; DEmiRNA: differentially expressed micro RNAs; high-exh, high-exhaustion tertile; BH: Benjamini-Hochberg; FDR: false discovery rate

Direction	miRNA ID	log_2_FC	FDR (BH-adjusted p-value)	High-exh presence
Up	hsa-miR-155-5p	+1.443	1.79e-06	100.0%
	hsa-miR-1976	+0.932	1.79e-06	100.0%
	hsa-miR-142-5p	+1.455	1.57e-05	100.0%
	hsa-miR-4772-3p	+1.397	2.14e-05	100.0%
	hsa-miR-342-5p	+1.134	5.50e-05	100.0%
	hsa-miR-223-5p	+1.390	1.28e-04	97.4%
Down	hsa-miR-361-5p	-0.631	1.07e-03	100.0%
	hsa-miR-125a-5p	-0.766	1.60e-02	100.0%
	hsa-miR-506-3p	-2.344	1.67e-02	92.1%
	hsa-miR-324-3p	-0.638	2.39e-02	100.0%

Screening 21,111 candidate miRNA-mRNA pairs identified 326 database-supported inverse interactions, linking 25 DEmiRNAs to 303 mRNA targets. One of the 26 initially identified DEmiRNAs did not meet the retention criteria and was excluded from the final network. The network density was 0.043, with the miR-146 and miR-200 families contributing 73 (22.4%) and 38 (11.7%) interactions, respectively. Of the 326 interactions, 263 were classified as active suppression (AS) and 63 as loss-of-repression (LoR). Experimental support from miRTarBase was available for only 15 edges (4.6%). Two focused LoR associations linked downregulated miRNAs to established exhaustion-related genes. hsa-miR-324-3p was lower in the high-exhaustion group (log_2_FC = -0.64, FDR = 0.024) and inversely associated with PDCD1 (rho = -0.307, edge FDR = 0.020); hsa-miR-506-3p was also lower (log_2_FC = -2.34) and inversely associated with EGR2 (rho = -0.383, edge FDR = 0.002). After covariate adjustment, miR-324-3p-PDCD1 had beta = -0.128 (p = 0.067, FDR = 0.086), and miR-506-3p-EGR2 had beta = -0.119 (p = 0.502, FDR = 0.502), consequently, neither met the adjusted-support criterion (Table 2).

**Table 2:** Focused miRNA-mRNA axes and their covariate-adjusted classification. Network rho and edge FDR were calculated in the 115-sample paired dataset. a: Did not meet the adjusted-support criterion. Database support: ***miRDB and miRTarBase; **miRTarBase; *miRDB The adjusted-support criterion required beta < 0, nominal p ≤ 0.05, and BH FDR ≤ 0.10 across the nine focused models. AS: active suppression; LoR: loss-of-repression; BH: Benjamini-Hochberg; FDR: false discovery rate

miRNA	mRNA	Directional class	Network rho	Network edge FDR	Adjusted beta (FDR)
miR-155-5p	MEIS1***	AS	-0.470	2.11e-05	-0.266 (0.064)
miR-142-3p	NR2F6***	AS	-0.412	4.66e-04	-0.211 (0.064)
miR-146b-3p	ITPKB**	AS	-0.388	0.001	-0.244 (0.071)
miR-506-3p	EGR2*	LoR	-0.383	0.002	-0.119 (0.502)^a^
miR-146b-3p	MAVS**	AS	-0.373	0.002	-0.204 (0.092)^a^
miR-324-3p	PDCD1**	LoR	-0.307	0.020	-0.128 (0.086)^a^
miR-155-5p	FOXO3**	AS	-0.305	0.020	-0.354 (0.003)
miR-200c-3p	ZEB2***	AS	-0.305	0.021	-0.306 (0.003)
miR-200c-3p	PIK3CA*	AS	-0.301	0.023	-0.260 (0.041)

Active suppression axes

**Figure 2:**
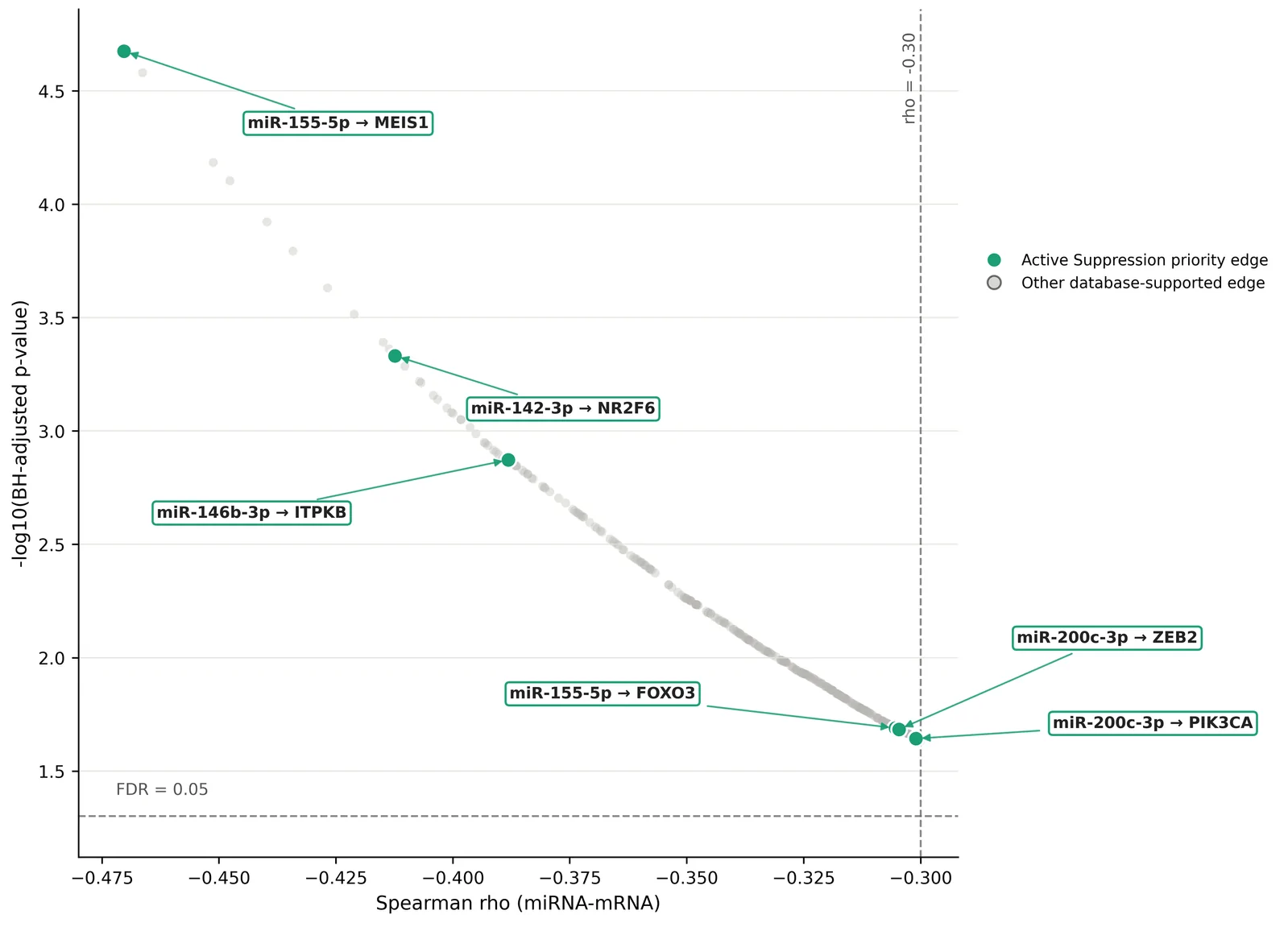
Spearman's correlation landscape of exhaustion-anchored miRNA-mRNA regulatory edges.

The AS category comprised 263 edges involving 20 upregulated DEmiRNAs. Six of the nine focused axes met the adjusted-support criterion, and all six were AS-classified. After adjustment, the miR-155-5p hub retained both of its strongest edges, MEIS1 and FOXO3, alongside miR-142-3p-NR2F6 and miR-146b-3p-ITPKB. Only miR-146b-3p-MAVS lost significance, though it remained inversely associated (β = -0.204, FDR = 0.092). Within the miR-200c-3p subnetwork, both ZEB2 (rho = -0.305, edge FDR = 0.021) and PIK3CA (rho = -0.301, edge FDR = 0.023) met the adjusted-support criterion (Figure 2).

Tripartite lncRNA-miRNA-mRNA ceRNA network

**Figure 3:**
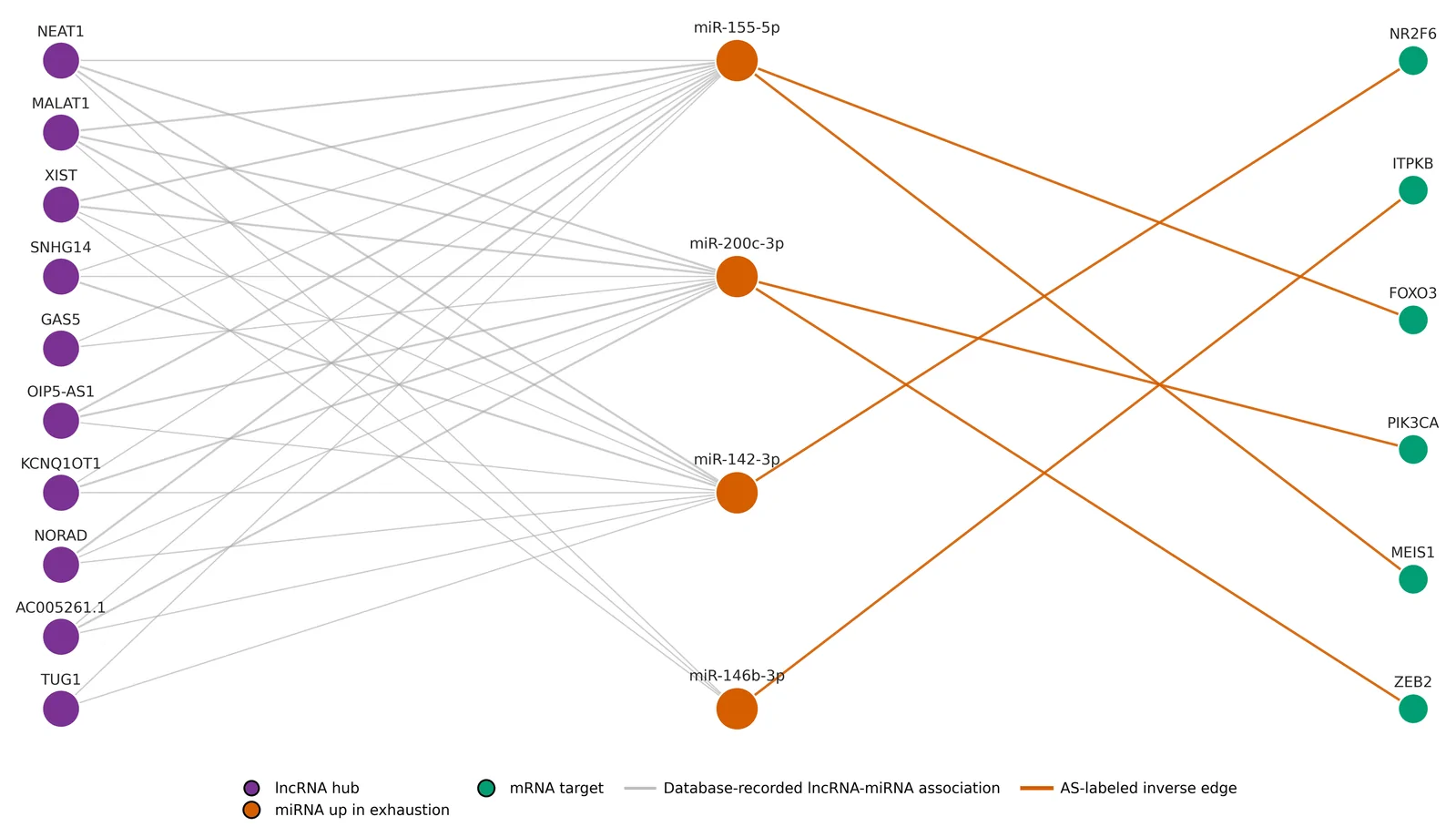
Priority lncRNA-miRNA-mRNA regulatory subnetwork associated with CD8+ T-cell exhaustion in melanoma. lncRNA: long non-coding RNA; miRNA: microRNA

The dual-database tripartite ceRNA network extension, incorporating 2,063 lncRNA-miRNA interactions (DIANA-LncBase v3 and ENCORI/starBase; 83 dual-source supported pairs) from 1,350 unique lncRNAs, yielded a tripartite graph of 1,679 nodes and 2,389 edges. The top topology-central lncRNA hubs were NEAT1, MALAT1, XIST, KCNQ1OT1, and SNHG14; all connected to priority mRNA targets through DEmiRNA intermediates (Figure 3).

Differential expression testing of the TCGA-SKCM lncRNA hubs yielded no statistically significant results after BH/FDR correction. The strongest trend was NORAD downregulation in high-exhaustion samples (log_2_FC = -0.398, raw *p *= 0.00849, FDR = 0.0849), which did not meet the prespecified significance threshold, indicating that the identified lncRNAs were not transcriptionally dysregulated in the CD8-enriched exhaustion model at the bulk level (Table 3).

**Table 3:** Top topology-central lncRNA hubs from the DIANA/ENCORI ceRNA database. Direction: ↑ up, ↓ down BH: Benjamini-Hochberg; FDR: false discovery rate; ENCORI: Encyclopedia of RNA Interactomes; ceRNA: competing endogenous RNA

lncRNA	Degree	Eigenvector	AS/LoR miRNAs	log_^2^_FC	FDR (BH-adjusted p)	Candidate mRNA targets
NEAT1 ↑	20	0.099	15/5	+0.367	0.9233	EGR2, FOXO3, ITPKB, MAVS, MEIS1, NR2F6, PDCD1, PIK3CA, ZEB2
MALAT1 ↑	19	0.099	14/5	+0.368	0.6864	EGR2, FOXO3, ITPKB, MAVS, MEIS1, NR2F6, PDCD1, PIK3CA, ZEB2
XIST ↑	18	0.099	14/4	+0.457	0.9742	EGR2, FOXO3, ITPKB, MAVS, MEIS1, NR2F6, PDCD1, PIK3CA, ZEB2
KCNQ1OT1 ↓	15	0.091	12/3	-0.173	0.9962	FOXO3, MAVS, MEIS1, NR2F6, PDCD1, PIK3CA, ZEB2
SNHG14 ↓	15	0.089	11/4	-0.251	0.9742	EGR2, FOXO3, MAVS, MEIS1, NR2F6, PDCD1, PIK3CA, ZEB2
OIP5-AS1 ↓	15	0.088	11/4	-0.016	0.9962	FOXO3, MEIS1, NR2F6, PDCD1, PIK3CA, ZEB2
NORAD ↓	14	0.087	12/2	-0.398	0.0849	FOXO3, MAVS, MEIS1, NR2F6, PDCD1, PIK3CA, ZEB2
TUG1 ↓	12	0.083	8/4	-0.098	0.6864	FOXO3, MAVS, MEIS1, NR2F6, PDCD1
AC005261.1 ↓	12	0.077	8/4	-0.245	0.5009	FOXO3, MAVS, MEIS1, NR2F6, PDCD1, PIK3CA, ZEB2
GAS5 ↓	10	0.074	6/4	-0.829	0.1429	EGR2, FOXO3, MAVS, MEIS1, PDCD1, PIK3CA, ZEB2

Continuous exhaustion-score and covariate-adjusted robustness analyses

For all 326 edge records, the direction of each connected miRNA's correlation with the continuous exhaustion score agreed with its grouped differential-expression label. In the nine complete-case HC3 models (n = 114), six axes met the adjusted-support criterion and all six were AS-classified. miR-506-3p-EGR2, miR-146b-3p-MAVS, and miR-324-3p-PDCD1 did not meet the criterion. Quartile-extreme testing supported six axes; all nine leave-one-out ranges remained inverse; the independent-signature gate supported five; and the tumor-only analysis supported seven. The tumor-only DEmiRNA analysis retained 25 of 26 miRNAs, with hsa-miR-584-5p falling below the full definition (Table 4).

**Table 4:** Covariate-adjusted and sensitivity results for nine focused miRNA-mRNA axes. *Meets the adjusted-support criterion (adjusted β < 0, nominal p ≤ 0.05, BH FDR ≤ 0.10). Q: quartile criterion; T: tumor-only criterion; Alt: independent-signature gate; LOO: leave-one-out ρ range across folds (Y (yes)/N (no)) indicates whether each sensitivity criterion was met; BH q-value: Benjamini-Hochberg false discovery rate-adjusted p-value; BH FDR: Benjamini-Hochberg false discovery rate; CI: confidence interval

miRNA-mRNA axis	Adjusted β (95% CI)	Adjusted p-value (BH q-value)	Partial Spearman ρ (BH q-value)	Sensitivity (Q/T/Alt)	LOO ρ range
miR-155-5p-MEIS1^*^	-0.266 (-0.513, -0.018)	0.035 (0.064)	-0.400 (9.52 × 10^-5^)	Y/Y/Y	-0.498 to -0.457
miR-142-3p-NR2F6^*^	-0.211 (-0.399, -0.022)	0.028 (0.064)	-0.204 (0.030)	Y/Y/Y	-0.436 to -0.397
miR-146b-3p-ITPKB^*^	-0.244 (-0.485, -0.003)	0.047 (0.071)	-0.250 (0.012)	Y/Y/Y	-0.422 to -0.373
miR-506-3p-EGR2	-0.119 (-0.466, 0.228)	0.502 (0.502)	-0.322 (0.001)	Y/Y/Y	-0.419 to -0.369
miR-146b-3p-MAVS	-0.204 (-0.434, 0.025)	0.081 (0.092)	-0.242 (0.012)	Y/Y/Y	-0.409 to -0.358
miR-324-3p-PDCD1	-0.128 (-0.266, 0.009)	0.067 (0.086)	-0.216 (0.024)	N/Y/N	-0.336 to -0.290
miR-155-5p-FOXO3^*^	-0.354 (-0.560, -0.149)	7.23 × 10^-4^ (0.003)	-0.321 (0.001)	Y/N/N	-0.329 to -0.288
miR-200c-3p-ZEB2^*^	-0.306 (-0.482, -0.130)	6.44 × 10^-4^ (0.003)	-0.249 (0.012)	N/Y/N	-0.326 to -0.286
miR-200c-3p-PIK3CA^*^	-0.260 (-0.466, -0.053)	0.014 (0.041)	-0.242 (0.012)	N/N/N	-0.330 to -0.283

Exploratory clinical association analyses

**Figure 4:**
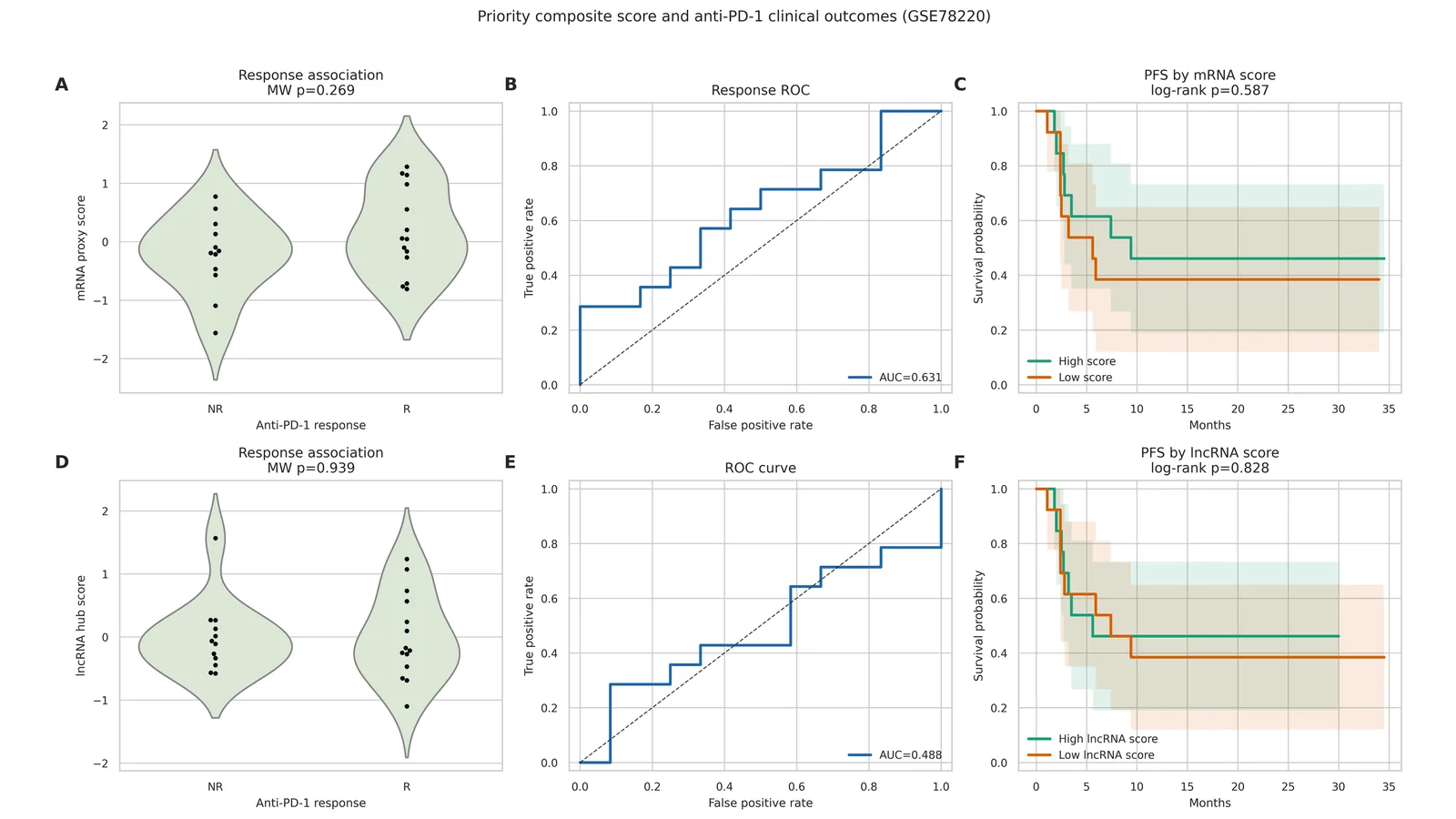
Exploratory GSE78220 clinical association. (A-C) Priority-target mRNA proxy score by response, ROC curve, and survival stratification. (D-F) The lncRNA hub score analyses. ROC: receiver operating characteristic; lncRNA: long non-coding RNA

GSE78220 included 26 evaluable pretreatment patients (14 responders and 12 non-responders). The target-mRNA proxy showed modest but statistically non-significant response discrimination (ROC-AUC = 0.631; bootstrap 95% confidence interval (CI) = 0.406-0.835; Mann-Whitney U = 106.0, p = 0.269). Median scores were +0.049 among responders and -0.176 among non-responders. Median-split survival curves did not separate PFS (log-rank p = 0.587) or overall survival (p = 0.725) (Figure 4A-4C). The four-lncRNA score was near chance (AUC = 0.488; Mann-Whitney p = 0.939) and was weakly oriented toward higher values in non-responders (Figure 4D-4F).

**Figure 5:**
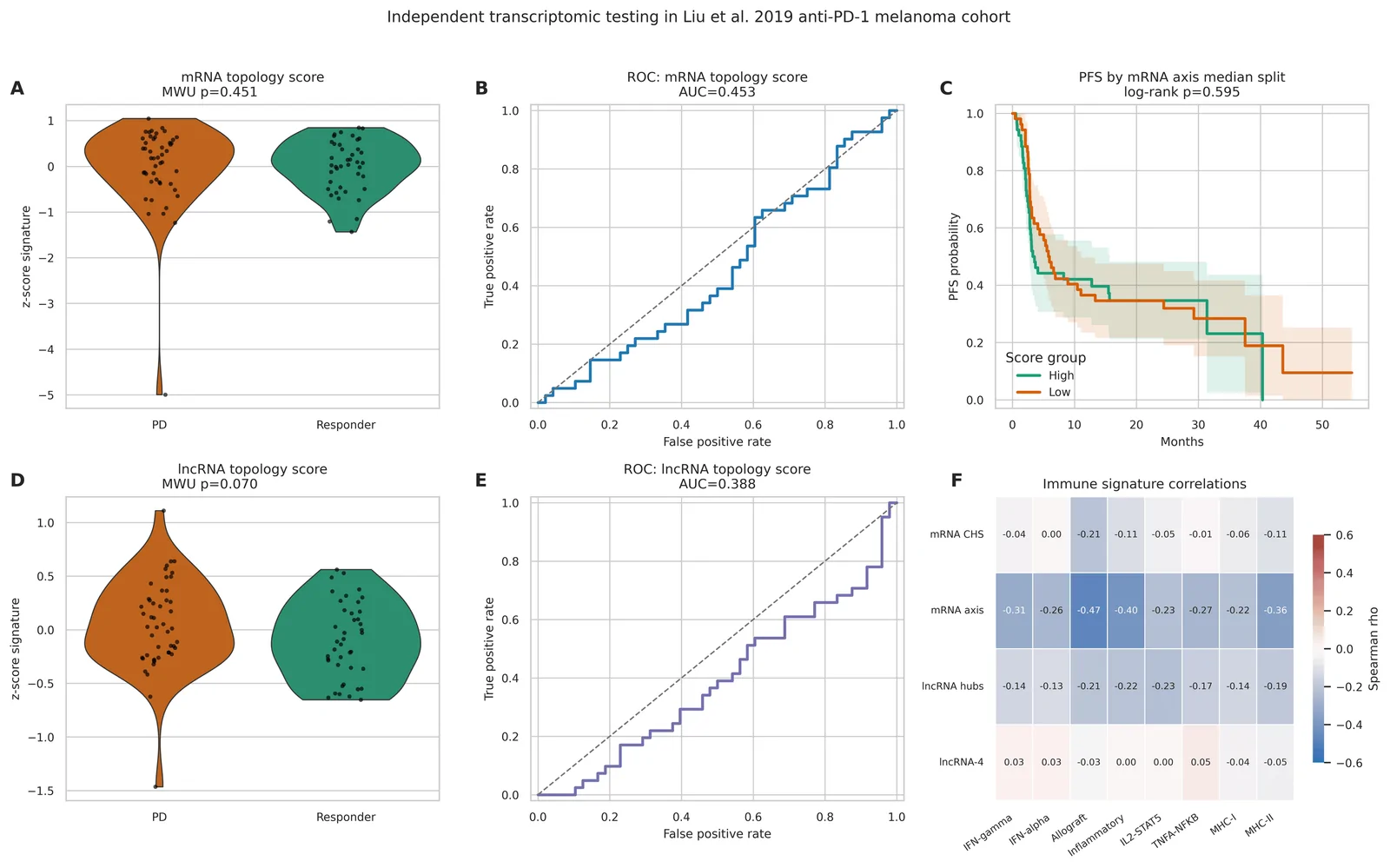
Independent transcriptomic testing in the Liu et al. 2019 pre-PD-1 subset. (A-B) The connected mRNA topology score by response and ROC curve. (C) PFS by the function-minus-exhaustion mRNA axis. (D-E) The lncRNA topology score. (F) Spearman's correlations between each transcriptomic score and eight immune-signature gene sets: interferon-gamma response, interferon-alpha response, allograft rejection, inflammatory response, IL-2-STAT5 signaling, TNF-alpha signaling via NF-kappa B, MHC class I, and MHC class II. PFS: progression-free survival; ROC: receiver operating characteristic; IL: interleukin; TNF: tumor necrosis factor; NF: nuclear factor; MHC: major histocompatibility complex; lncRNA: long non-coding RNA

The Liu et al. 2019 cohort provided 121 RNA-seq samples with clinical overlap. The prespecified primary analysis used the 104 Pre-PD1 biopsy samples, including 48 PD, 27 PR, 14 CR, 13 SD, and 2 MR cases. Because miRNA-seq was unavailable, testing was restricted to downstream transcriptomic scores rather than direct DEmiRNA abundance or miRNA-mRNA edge replication. For the primary CR/PR versus PD endpoint (n = 89), no tested score showed response discrimination in the expected direction: connected mRNA topology score (AUC = 0.453, Mann-Whitney p* *= 0.451; Figure 5A-5B), function-minus-exhaustion mRNA axis (AUC = 0.438, p = 0.313; its PFS analysis is shown in Figure 5C), lncRNA topology hub score (AUC = 0.388, p = 0.0695; Figure 5D-5E), and four-lncRNA priority score (AUC = 0.413, p = 0.158, not plotted in Figure 5). The corresponding correlations between the four transcriptomic scores and the eight immune-signature gene sets are shown in Figure 5F.

Median-split survival analyses were non-significant for the mRNA topology score (PFS log-rank p = 0.449; OS p = 0.792) and the function-minus-exhaustion mRNA axis (PFS p = 0.595; OS p = 0.535). The lncRNA topology score showed a nominal univariate overall survival (OS) Cox association (hazard ratio (HR) = 1.44, p = 0.0065), but this was not retained after covariate adjustment (p = 0.131) and did not coincide with response discrimination. The function-minus-exhaustion mRNA axis showed inverse correlations with immune activation signatures, including allograft rejection (ρ = -0.473, p = 1.3 x 10^-5^), inflammatory response (ρ = -0.404, p = 3.4 x 10^-4^), major histocompatibility complex (MHC)-II (ρ = -0.365, p = 0.0015), and interferon-gamma response (ρ = -0.312, p = 0.0103). These correlations support immune-context relevance but not clinical predictive validity (Figure 5F).

## Discussion

This study identifies a topology-informed miRNA-mRNA regulatory network associated with CD8/exhaustion-enriched melanoma bulk tumors. The LoR/AS framework is useful because it distinguishes inverse correlations according to whether the miRNA rises or falls with exhaustion. It remains an operational classification rather than a mechanistic conclusion. Single-cell studies of melanoma have demonstrated progenitor-like, proliferative, dysfunctional, and terminally exhausted CD8+ states within the same tumor ecosystem [23]. Accordingly, the network is best viewed as a bulk-tissue experimental prioritization map. Because the discovery dataset consisted of retrospective bulk tumor transcriptomes, every edge was treated as an association rather than evidence of direct binding or repression. The analysis cannot localize a signal to CD8+ T cells, establish protein-level effects, or demonstrate ceRNA competition. The resulting axes are consequently presented as experimentally testable hypotheses.

The miR-324-3p-PDCD1 association illustrates the need for restraint. PDCD1 expression is governed by chronic antigen signaling, TOX/NR4A-centered transcriptional programs, and exhaustion-associated chromatin remodeling [7,24]. An inverse bulk correlation with miR-324-3p is compatible with an additional post-transcriptional contribution but cannot demonstrate one. miR-28 has altered PD-1 and exhaustion-associated readouts in a separate experimental system [25], providing precedent for miRNA involvement without validating miR-324-3p. In the present data, miR-324-3p-PDCD1 did not meet the adjusted-support, quartile, or independent-gate criteria, although it remained supported in the tumor-only sensitivity analysis. Direct 3'UTR and perturbation experiments would be required before assigning this axis a regulatory role.

A similar distinction applies to miR-506-3p-EGR2. Wagle et al. showed that antigen-driven EGR2 expression is sustained in progenitor exhausted CD8+ T cells and helps stabilize exhaustion-associated transcriptional and epigenetic programs [26], making the proposed relationship biologically plausible. Yet the adjusted coefficient was attenuated (beta = -0.119, p = 0.502, FDR = 0.502), despite an inverse partial-rank estimate and supportive results in some sensitivity analyses. Biological coherence cannot substitute for statistical independence, and this association remains a context-dependent hypothesis rather than an established LoR mechanism.

Among the AS-classified associations, miR-155-5p was a prominent node. miR-155 participates in inflammatory T cell programs [10] and can enhance antitumor CD8+ T cell fate through Phf19/PRC2-linked epigenetic reprogramming [27]. FOXO3, in turn, regulates CD8+ memory through T-cell-intrinsic mechanisms [28]. These observations make the miR-155-FOXO3 and miR-155-MEIS1 axes experimentally tractable, but they do not show that miR-155 directly targets either transcript in exhausted melanoma lymphocytes. Nor should miR-155 upregulation be equated automatically with loss of CD8 function; it may reflect sustained activation, cellular abundance, or context-dependent adaptation. This also helps explain why the clinical mRNA proxy score showed a weak positive direction in responders: miR-155-associated topology may partly mark inflamed, target-bearing tumors rather than terminal immune failure.

The remaining adjusted-supported axes also require cell-context resolution. The miR-200c-3p-ZEB2 relationship is compatible with the established role of ZEB-family factors in differentiation and phenotype-state transitions, but a bulk melanoma signal may arise from malignant, stromal, or immune cells. miR-146 family biology is consistent with feedback control of inflammatory and innate signaling through NF-kappa B-associated pathways [11], whereas the candidate edges here involve miR-146b-3p, a passenger-strand species with less direct experimental evidence than the miR-146a and miR-146b-5p literature. These associations are therefore better regarded as convergent hypotheses than as validated pathways.

The lncRNA extension broadens this hypothesis space but does not establish a ceRNA mechanism. Earlier melanoma studies linked reconstructed ceRNA networks to progression or prognosis [12,13], whereas the present network was restricted to exhaustion-associated miRNAs. Genuine ceRNA competition requires co-expression in the same cell, compatible subcellular localization, sufficient transcript abundance, appropriate binding-site stoichiometry, and demonstrable competition [29]. The 29,274 relational chains are consequently an inclusive set of candidate paths. Neither high topology centrality nor inclusion among the 50 displayed chains demonstrates sponging, and none of the tested lncRNA hubs was differentially expressed after BH correction.

The clinical findings are best interpreted as a negative translational result. Hugo et al. described innate anti-PD-1 resistance as a multifactorial program involving mesenchymal, wound-healing, angiogenic, and immunosuppressive features [15]. Liu et al. likewise integrated genomic, antigen-presentation, immune, and clinical variables rather than relying on a single transcript score [18]. Broader network modeling has reached a similar conclusion: response prediction depends on the surrounding gene-network and therapeutic context [30]. Thus, clinical response to PD-1 blockade is best treated as a multifactorial phenotype rather than as the output of a single miRNA-mRNA topology score.

Within this context, the present clinical findings are appropriately negative and hypothesis-generating. The GSE78220 mRNA proxy score produced a modest ROC-AUC of 0.631, which is within the broad range of modest transcriptomic immune biomarkers reported in melanoma, but its statistical uncertainty and proxy construction prevent biomarker interpretation. Median scores were higher in responders than non-responders, but survival separation was not significant for PFS or OS. The lncRNA hub score performed no better than chance and was directionally inverted. Independent testing in Liu et al.'s pre-PD-1 cohort was also non-significant. These results argue against clinical utility of the current score and support a narrower interpretation that the topology captures regulatory hypotheses related to immune activation/exhaustion biology, but it does not yet define a deployable anti-PD-1 response biomarker.

Taken together, the data favor a distributed model rather than a single exhaustion-controlling axis. Chronic antigen stimulation can induce immune miRNAs while simultaneously reshaping transcription, chromatin, and cellular composition. In that setting, upregulated immune miRNAs such as miR-155-5p may reflect sustained antigen stimulation and tumor-infiltrating lymphocyte (TIL) persistence, producing both beneficial and suppressive downstream effects depending on target context. The predominance of AS-classified axes after adjustment is therefore a prioritization result, not proof that miRNA induction causes CD8 dysfunction. Downregulation of selected miRNAs, such as miR-324-3p and miR-506-3p, may release inhibitory-receptor or exhaustion-transcription-factor targets, increasing the stability of exhaustion-associated states. Likewise, topology-central mRNAs may mark convergence points where multiple miRNAs tune survival, interferon, inhibitory receptor, and differentiation programs rather than acting as single master regulators. Clinical response to PD-1 blockade additionally depends on antigen presentation, tumor genotype, stromal exclusion, myeloid suppression, the balance of progenitor and terminally exhausted T cells, disease burden, and treatment history, explaining why the downstream composite scores had limited discrimination.

Several limitations follow directly from the study design. The network was selected from inverse correlations in retrospective bulk data and then examined in the same cohort, so adjusted and sensitivity analyses assess robustness rather than independent confirmation. Database representation does not guarantee direct binding, and no protein, perturbation, or cell-specific measurements were available. Residual spatial, treatment, tumor-subtype, and cell-state confounding remains. Certain factors limited the clinical analyses. Neither GSE78220 nor the independent DFCI melanoma cohort included matched miRNA measurements; therefore, testing relied on downstream mRNA or lncRNA proxy scores rather than direct replication of the original miRNA-mRNA network. GSE78220 was small (n = 26), did not support reliable multivariable modeling, and used PFS annotations curated from a published supplement rather than directly from GEO. In the DFCI cohort, Cox models incorporated available nonsynonymous mutation burden, tumor purity, ploidy, prior CTLA-4 exposure, and M stage; however, neither cohort provided a complete set of serum lactate dehydrogenase (LDH), Eastern Cooperative Oncology Group (ECOG) performance status, disease-burden, treatment-history, melanoma-subtype, and antigen-presentation covariates.

Future work should therefore prioritize cell-specific validation before clinical extrapolation. Experimental work should proceed in cell-resolved systems. If the miR-324-3p-PDCD1 or miR-506-3p-EGR2 hypotheses are pursued, wild-type and seed-mutant 3'UTR reporters should be combined with miRNA mimic/inhibitor perturbation in chronically stimulated primary human CD8+ T cells, followed by qPCR and PD-1 or EGR2 protein measurement. The miR-155-5p-FOXO3/MEIS1 axes require analogous perturbation and protein assays together with assessment of the Phf19/PRC2 context [27]. Candidate ceRNA paths require localization, AGO/CLIP support, abundance and stoichiometry measurements, and rescue experiments. The most informative replication design would pair small-RNA, lncRNA, mRNA, and protein measurements in sorted or single-cell melanoma CD8+ tumor-infiltrating lymphocytes with T cell receptor and spatial annotation.

## Conclusions

This in silico study characterized the miRNA regulatory landscape associated with CD8⁺ T-cell exhaustion in melanoma using a biologically guided LoR/AS framework. The analysis identified miR-324-3p-PDCD1, miR-506-3p-EGR2, and miR-155-5p-FOXO3/MEIS1 as prioritized candidate regulatory axes and highlighted NEAT1, MALAT1, and XIST as potential lncRNA hubs within a putative ceRNA network. However, all reported associations are correlative and derived from bulk tumor transcriptomic data; therefore, they do not establish causality or T cell-intrinsic regulatory mechanisms. Exploratory clinical analyses were non-significant and should be regarded as hypothesis-generating rather than as evidence of predictive utility. Overall, the study provides a framework for future experimental validation and prospective clinical investigation of miRNA-mediated regulation of T-cell exhaustion in melanoma.

## Appendices

**Table 5:** The 26 DEmiRNA list. FDR: false discovery rate; BH: Benjamini-Hochberg; exh: exhaustion

miRNA ID	Log_^2^_FC	Raw p-value	Mean high exh	Mean low exh	FDR BH	Direction	High exh presence pct
hsa-miR-155-5p	1.443432764	9.063E-09	11.71961602	10.27618326	1.785E-06	Up in exhaustion	100
hsa-miR-1976	0.9322598	5.915E-09	5.377377895	4.445118095	1.785E-06	Up in exhaustion	100
hsa-miR-142-5p	1.455132199	1.197E-07	6.94313656	5.488004361	1.572E-05	Up in exhaustion	100
hsa-miR-4772-3p	1.396941379	2.168E-07	4.029185136	2.632243758	2.136E-05	Up in exhaustion	100
hsa-miR-342-5p	1.134132552	6.982E-07	5.503355827	4.369223275	5.502E-05	Up in exhaustion	100
hsa-miR-223-5p	1.390200431	2.267E-06	3.182986353	1.792785922	1.276E-04	Up in exhaustion	97.36842105
hsa-miR-625-3p	1.010417945	2.151E-06	9.376695316	8.366277372	1.276E-04	Up in exhaustion	100
hsa-miR-150-5p	1.89095532	2.917E-06	12.48023108	10.58927576	1.437E-04	Up in exhaustion	100
hsa-miR-142-3p	1.638541421	4.044E-06	11.4914207	9.852879277	1.593E-04	Up in exhaustion	100
hsa-miR-342-3p	1.20983304	3.663E-06	9.667198278	8.457365239	1.593E-04	Up in exhaustion	100
hsa-miR-361-5p	-0.630674628	2.999E-05	8.852933952	9.48360858	1.074E-03	Down in exhaustion	100
hsa-miR-223-3p	1.135308869	5.665E-05	8.840231957	7.704923089	1.860E-03	Up in exhaustion	100
hsa-miR-146b-3p	0.81573745	6.316E-05	8.257644142	7.441906692	1.914E-03	Up in exhaustion	100
hsa-miR-200c-3p	1.12108518	1.623E-04	6.847182413	5.726097233	4.567E-03	Up in exhaustion	100
hsa-miR-3614-5p	0.85639884	4.811E-04	3.457759854	2.601361014	1.264E-02	Up in exhaustion	100
hsa-miR-7-1-3p	0.81810584	6.675E-04	5.164096727	4.345990886	1.602E-02	Up in exhaustion	100
hsa-miR-125a-5p	-0.76574951	6.911E-04	9.430396893	10.1961464	1.602E-02	Down in exhaustion	100
hsa-miR-766-3p	0.684696826	8.053E-04	5.25318328	4.568486454	1.672E-02	Up in exhaustion	100
hsa-miR-506-3p	-2.344082806	8.065E-04	5.294134374	7.63821718	1.672E-02	Down in exhaustion	92.10526316
hsa-miR-6842-3p	0.606861899	8.681E-04	2.860056617	2.253194717	1.710E-02	Up in exhaustion	100
hsa-miR-324-3p	-0.637626423	1.276E-03	6.111312493	6.748938916	2.394E-02	Down in exhaustion	100
hsa-miR-942-5p	0.526891904	2.064E-03	4.508715209	3.981823305	3.696E-02	Up in exhaustion	100
hsa-miR-511-5p	0.849842407	2.254E-03	5.278255801	4.428413393	3.861E-02	Up in exhaustion	100
hsa-miR-146b-5p	0.857936698	2.626E-03	10.24015763	9.382220928	4.104E-02	Up in exhaustion	100
hsa-let-7e-3p	-0.741637005	2.676E-03	3.441956464	4.183593469	4.104E-02	Down in exhaustion	100
hsa-miR-584-5p	-0.897994441	2.714E-03	10.2949398	11.19293424	4.104E-02	Down in exhaustion	100
